# The 4‐Strand Hamstring Overlap Link for All‐Inside Anterior Cruciate Ligament Reconstruction: Graft Preparation Technique

**DOI:** 10.1002/atn2.70077

**Published:** 2026-07-14

**Authors:** Yan Yan, Chen Chen, Yang Tang, Di Wu, Ying‐Ming Wang, Qi‐Chun Zhao

**Affiliations:** ^1^ Department of Orthopaedics The First Affiliated Hospital of USTC Division of Life Sciences and Medicine University of Science and Technology of China Hefei Anhui China; ^2^ Department of Geriatric Dentistry NMPA Key Laboratory for Dental Materials National Engineering Laboratory for Digital and Material Technology of Stomatology Peking University School and Hospital of Stomatology Beijing China

## Abstract

Anterior cruciate ligament (ACL) injury is among the most prevalent sports‐related knee injuries. Arthroscopic ACL reconstruction (ACLR) is the standard minimally invasive surgical treatment for ACL injuries. The all‐inside technique, as a significant recent innovation in ACLR, has become an important clinical option due to its advantages of reduced invasiveness, stronger graft constructs, and less early postoperative pain. However, a consensus on the optimal tendon preparation technique for all‐inside ACLR has yet to be established. This article details an overlapping suture graft preparation technique for a 4‐strand autologous hamstring tendon graft, augmented with an internal brace and specifically designed for all‐inside ACLR. This technique maximizes the utilization of autologous tendon tissue, ensures uniform tension distribution across all strands, eliminates midsubstance gaps, and facilitates functional ACL restoration while promoting graft‐bone integration.

## Graphical abstract

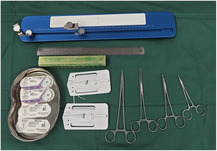

**VIDEO 1:** This video shows the preparation of a 4‐strand autologous hamstring tendon graft using an overlapping suture technique for all‐inside anterior cruciate ligament reconstruction. It begins with harvesting and cleaning the semitendinosus tendon, followed by measuring its quadrupled diameter to determine graft suitability. The core “overlap‐linkage” technique is then detailed: creating a tendon loop with a 4‐0 absorbable suture, folding it around a suspensory button loop to form a 4‐strand construct, and adjusting the overlap to achieve a final graft length of 58 to 65 mm. After pretensioning on a graft station to ensure uniform strand tension, the midportion is secured with a circumferential 4‐0 absorbable suture to eliminate gaps. The femoral and tibial ends are independently whipstitched with a #2 ORTHOCORD suture over 20 and 18 mm, respectively, serving both for fixation and as arthroscopic depth markers. An internal brace is incorporated by passing a #2 ORTHOCORD suture through the femoral button and routing it internally through the graft. The technique emphasizes precise graft length modulation, uniform tension distribution, and integrated internal bracing to construct a graft for all‐inside anterior cruciate ligament reconstruction. Video content can be viewed at https://doi.org/10.1002/atn2.70077.

Anterior cruciate ligament (ACL) injuries predominantly result from noncontact mechanisms in athletic populations aged 15 to 40 years, commonly during sports such as basketball, soccer, volleyball, and skiing. Approximately 3% of amateur athletes experience an ACL injury annually, a rate that rises to 15% among professional athletes.^1^ Women have a twofold to eightfold higher incidence of ACL injuries compared with men.^2^ In the USA, approximately 200,000 ACL injuries occur annually, with about 120,000 patients undergoing ACL reconstruction (ACLR) surgery each year.^3^

Given the high prevalence of ACL injuries and the persistent demand for improved outcomes, sports medicine physicians continuously refine surgical techniques and graft selection methods.^4^, ^5^ As a significant innovation in ACLR, the all‐inside technique has become an important clinical option owing to its advantages of reduced invasiveness, greater graft strength, and less early postoperative pain.^6^ Regarding graft choice, although allografts have shown good outcomes in some studies, they are associated with higher failure rates in younger, more active patients. Furthermore, ethical concerns regarding allograft procurement and potential disease transmission may also limit their application.^7^ The use of synthetic ligaments in ACLR has gained increasing acceptance in recent years. Literature reviews indicate that augmenting soft tissue grafts with a synthetic ligament can yield positive outcomes.^8^ When combined with promising scaffold technologies, next‐generation synthetic ACL grafts may offer considerable benefits as structural augmentations. A key limitation of synthetic ligaments, however, is their dependence on a sufficient residual native ACL for fixation.^9^ Consequently, autografts—particularly autologous hamstring tendon grafts—remain a good choice for ACLR.^5^

This article details an overlap graft preparation technique for a 4‐strand autologous hamstring tendon graft augmented with an internal brace, specifically designed for all‐inside ACLR. This technique employs an overlapping weaving method, whereby the length of the overlapped segment is adjusted to achieve the desired final graft length. The tendon is then pretensioned, and locked sutures are placed at both ends, with additional ligatures applied to eliminate gaps in the midsubstance of the graft. Finally, a reinforcing suture tape is incorporated into the construct to function as an internal brace. The principal advantages of this technique include maximizing the utilization of autograft tissue, ensuring uniform tension distribution across all strands, eliminating intragraft gaps, and facilitating anatomical ACL restoration while promoting graft‐bone healing.

## SURGICAL TECHNIQUE

The semitendinosus tendon was harvested from the medial aspect of the proximal tibia and cleared of adherent muscle tissue to obtain a clean graft. The diameter of the potential quadrupled graft was measured to determine whether concomitant harvest of the gracilis tendon was necessary. The prepared tendon was first precisely overlapped and sutured to create a closed‐loop construct. This looped tendon was then folded to form a quadruple‐stranded graft. The midportion of the graft was then gathered and secured with a fine‐gauge absorbable suture. Finally, under pretension, the femoral and tibial ends of the graft were whipstitched with a high‐strength suture for both fixation and length marking. An internal brace was incorporated to complete the graft preparation. The final graft was configured to a length of 58 to 65 mm and a diameter of 8 to 11 mm for all‐inside ACLR with suspensory button fixation on both the femoral and tibial sides. We describe this overlap graft preparation technique in this report and in Video 1.

### Graft Station Setup

**FIGURE 1:**
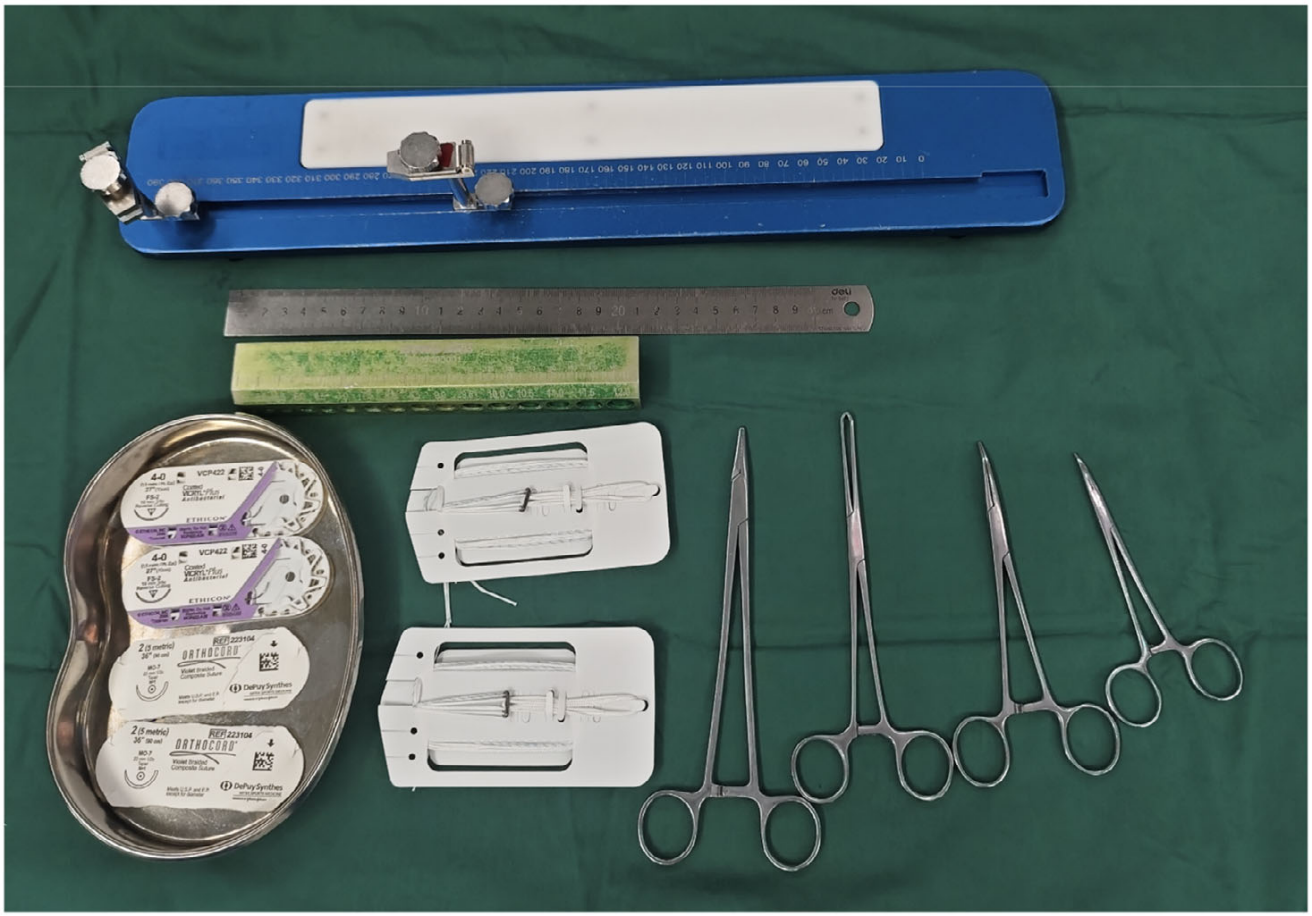
A graft preparation station was set up to prepare the 4‐strand ACL autograft construct. The suspensory button, high‐strength sutures, absorbable sutures, and all necessary surgical instruments were assembled and ready for use. (ACL, anterior cruciate ligament.)

The following equipment is used for this graft preparation technique: 2 buttons with looped sutures (#5 UHS Fiber, Delta Medical, China), 2 high‐strength looped sutures (#2 ORTHOCORD High Strength Suture, DePuy Synthes, USA); 2 4‐0 absorbable sutures (4‐0 coated VICRYL Plus, Ethicon, USA), and a graft preparation station (DePuy Synthes, USA) (Figure 1).

### Tendon Preparation

**FIGURE 2:**
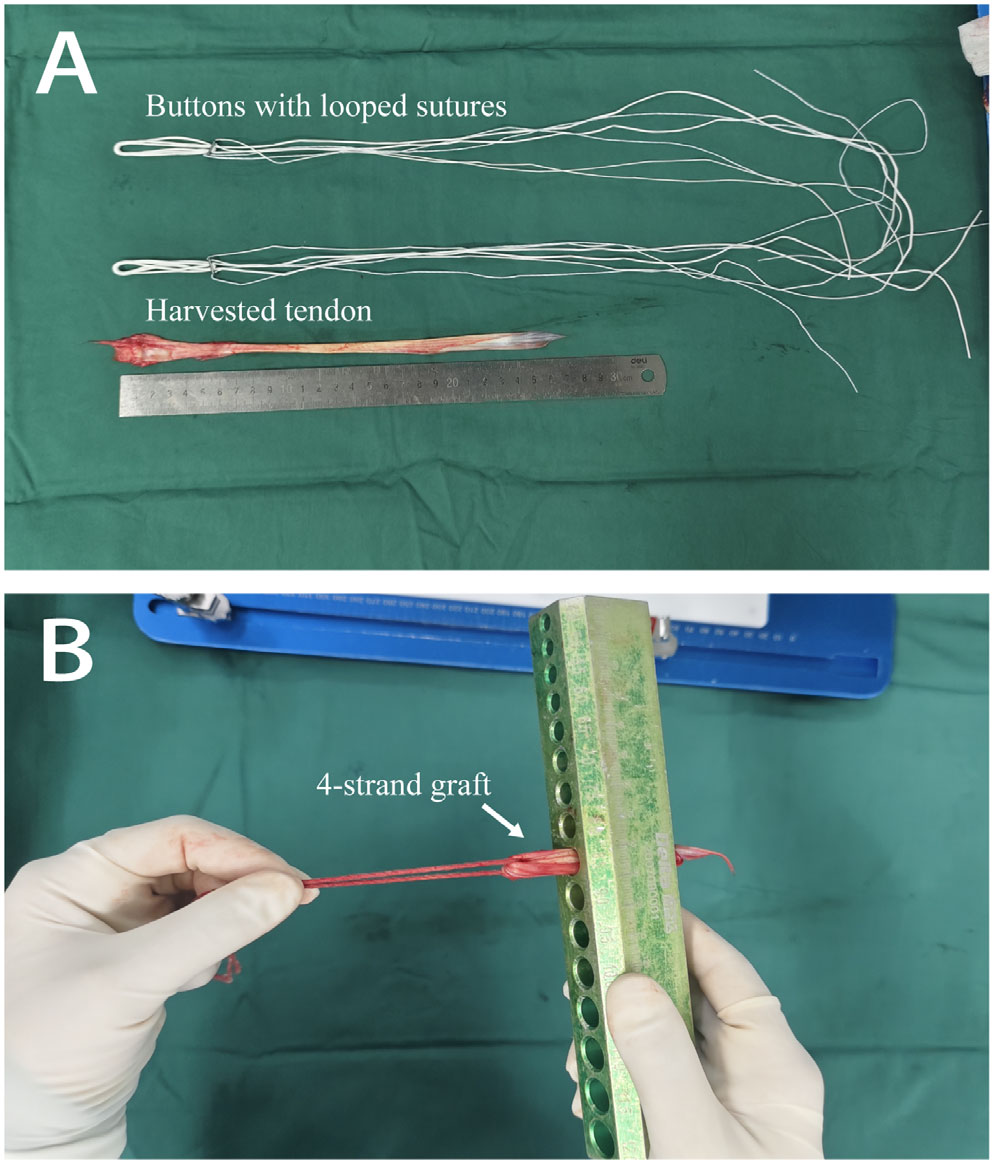
Graft preparation. (A) The harvested semitendinosus tendon is cleared of adherent muscle tissue to ensure a clean graft. (B) The diameter of the potential quadrupled tendon (white arrow) is measured to determine whether additional harvest of the gracilis tendon is required.

The semitendinosus tendon was harvested through a medial approach to the proximal tibia. The tendon was cleared of adherent muscle tissue, and the diameter of the potential quadrupled graft was measured to assess its suitability against a minimum 8 mm threshold. If the semitendinosus tendon alone was inadequate, the gracilis tendon was additionally harvested (Figure 2).

### Four‐Strand Overlap Linkage

**FIGURE 3:**
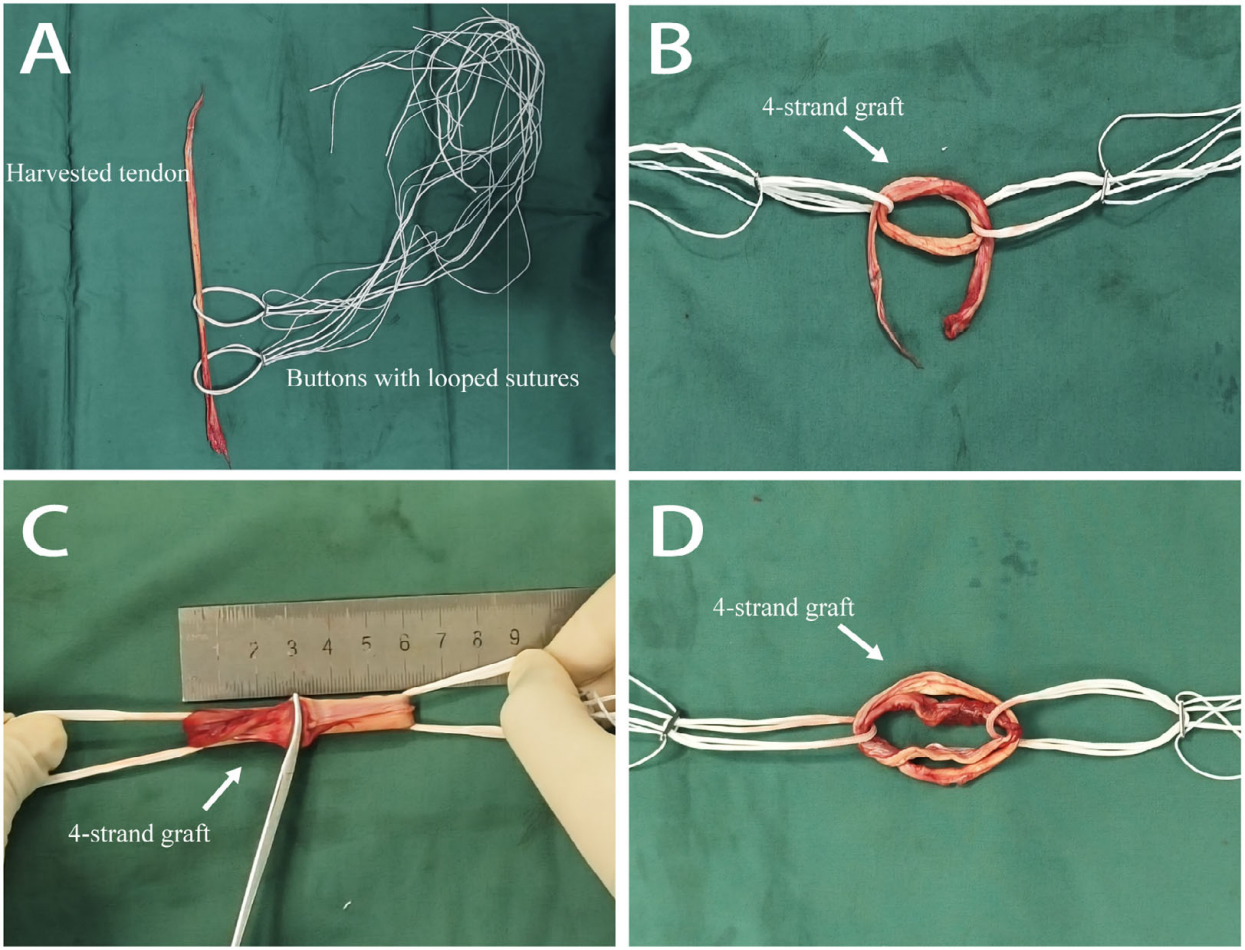
Four‐strand hamstring overlap linkage technique. (A) The tendon is passed through the loop of the suspensory button. (B) The tendon is folded around the loop to create a 4‐strand graft construct (white arrow). (C) The graft length is adjusted by modifying the overlap length of the tendon ends according to intraoperative requirements (white arrow). (D) Under tension, the overlapping tendon ends are sutured to form a continuous, looped configuration (white arrow).

1. The free tendon end is integrated with the main tendon body using a 4‐0 absorbable suture to maximize the integrity of the harvested graft.

2. The tendon is passed through the loop of the adjustable suspensory cortical button and wrapped twice around it. The 2 ends of the tendon are then folded back, and the overlapped portion is grasped with a small curved hemostat (Figure 3A,B).

3. Under uniform tension applied to all strands, the overlapped segment is repeatedly adjusted according to the surgeon's preference and the actual tendon length until the total graft length is controlled within the range of 58 to 65 mm (Figure 3C).

4. Subsequently, the folded portion is provisionally secured with a 4‐0 absorbable suture, stitching through exactly the amount of tissue involved in the fold (Figure 3D).

### Graft Pretention and Weaving

**FIGURE 4:**
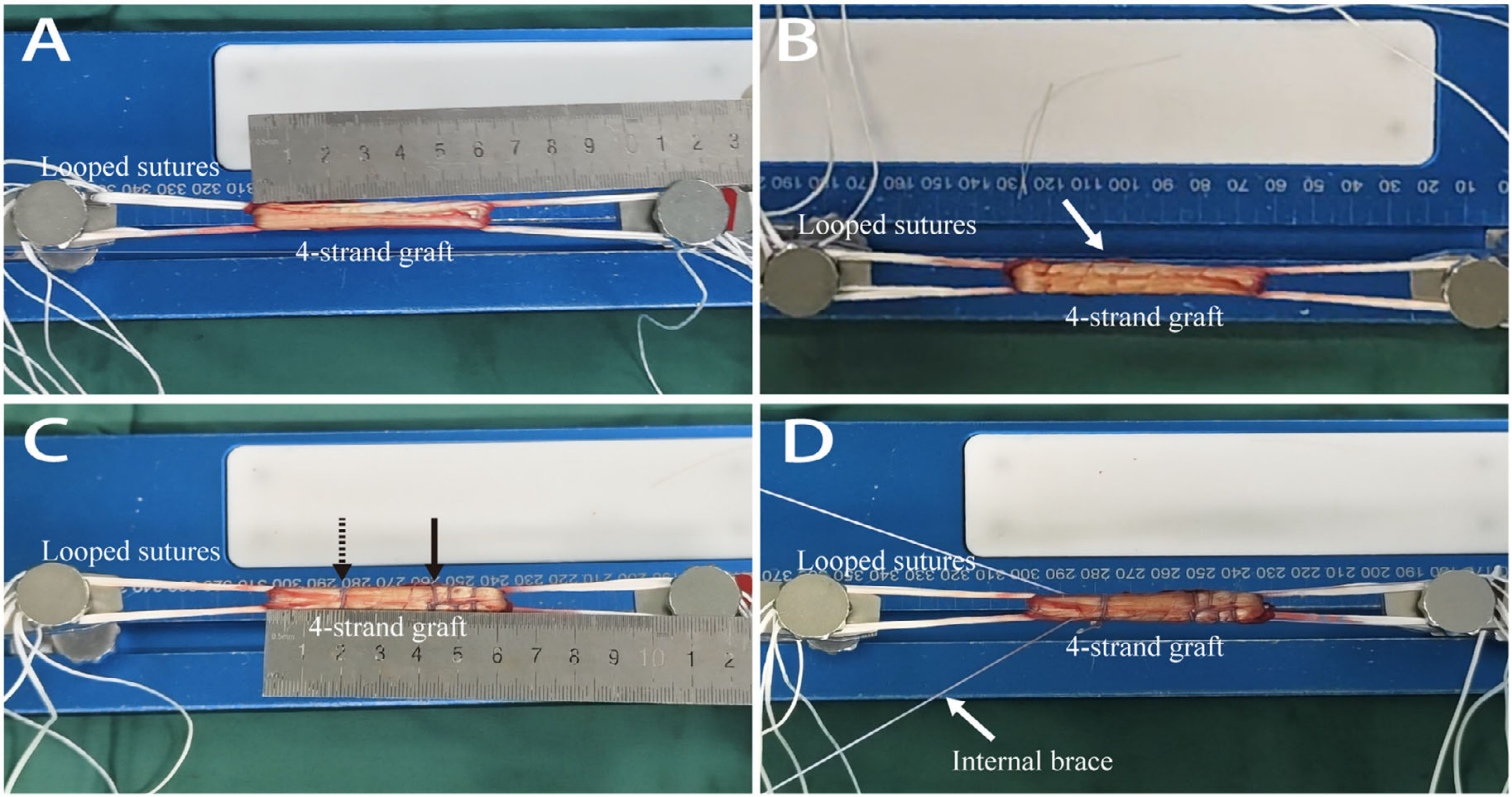
Graft tensioning and final preparation. (A) The graft is subjected to continuous pretensioning on the posts of a graft preparation station. (B) Under tension, the midportion of the graft is gathered and sutured with a 4‐0 absorbable suture (white arrow). (C) The femoral (20 mm, black dashed arrow) and tibial (18 mm, black arrow) ends of the graft are whipstitched with a #2 ORTHOCORD high‐strength suture. (D) An internal brace (white arrow) is created by passing a single #2 ORTHOCORD suture through the femoral button and routing its free ends internally through the graft.

1. Following initial overlapping suturing for graft formation, the tendon junction is positioned at the tibial side to prevent suture ends from protruding into the joint cavity. Simultaneously, sutures at the junction are adjusted toward the inner aspect of the tendon to minimize residual suture material within the bone tunnel. The looped ends of the preliminarily shaped graft are mounted onto the respective posts of an ACL graft preparation station for pretensioning, ensuring uniform tension across all strands (Figure 4A).

2. Under maintained tension, the midportion of the graft is gathered and secured with a 4‐0 absorbable suture to eliminate any visible gaps between the strands (Figure 4B). When placing this circumferential suture, care should be taken to visualize the needle path from the side of the graft to avoid penetrating the adjustable loop sutures.

3. Subsequently, the femoral and tibial ends of the graft are independently whipstitched using a #2 ORTHOCORD high‐strength suture (achieving final fixation at the overlapped segment). Each stitch must penetrate every tendon strand and circumferentially encompass the graft. The femoral end is sutured over a 20‐mm segment, corresponding to the approximate length of the femoral tunnel, while also serving as a depth marker. The tibial end is sutured over 18 mm to fully embed the suture within the tunnel, avoiding interference between the suture and healing of the ACL remnant to the graft (Figure 4C, Table 1).

**TABLE 1:** Pearls and Pitfalls of the Overlap Link Graft

**Pearls**	**Pitfalls**
**Tendon preparation** The tendon strands are sutured and integrated into the main tendon body to maximize the utilization of the harvested tendon	**Junction reposition** The junction is repositioned to the inner aspect of the graft to avoid compromising the tendon‐bone healing process
**Overlap link** Under pretension, the overlap length is adjusted to 58 to 65 mm, and the thinner ends are sutured in an overlapping manner. The overlap length may require iterative adjustments to meet the specific surgical requirements	**Midsubstance suturing** The suturing should not be excessively tight nor should the stitches be placed too close together, as this may compromise tendon vascularization When placing this circumferential suture, care should be taken to avoid penetrating the adjustable loop sutures
**Final fixation** Under continuous tension, whipstitching is performed on both ends of the graft while simultaneously creating markings at 18 to 20 mm from each terminus.	**Final fixation** The marking on the tibial side should not be excessively long, as this may interfere with integration to the anterior cruciate ligament remnant

4. Finally, a single #2 ORTHOCORD high‐strength suture is passed through the femoral button and looped back. The 2 free ends of the suture are routed internally through the graft and exit at its tibial side to function as an internal brace (Figure 4D).

### Graft Fixation

**FIGURE 5:**
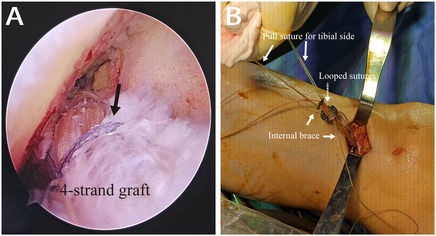
Graft fixation using the all‐inside ACLR technique. The patient is lying supine with the knee flexed at 90. Intra‐articular arthroscopic image of a right knee from the anterolateral viewing portal with a 30° arthroscope. (A) The graft is advanced into the femoral tunnel under arthroscopic guidance, using the preapplied high‐strength suture markings as a reference. (B) Final fixation is achieved by tensioning and securing the tibial suspensory button with the knee flexed at 30°. (ACLR, anterior cruciate ligament reconstruction.)

1. The femoral socket (20 mm in length) and tibial tunnel are prepared first. The graft is first introduced into the femoral tunnel and fixed with a suspensory button. Once the button passed beyond the premeasured depth of the narrow femoral tunnel (4.5 mm in diameter), it is flipped and seated against the lateral femoral cortex. The shortening loops are alternately tensioned to advance the graft into the femoral tunnel until the marking sutures are positioned precisely at the aperture of the femoral socket. The loops are then securely tied to the button using a knot pusher to achieve accurate fixation (Figure 5A).

2. Subsequently, the tibial end of the graft—with its attached loop, button traction suture, and internal brace sutures—is introduced into the tibial tunnel. To tension the graft, the button traction suture was vigorously tensioned while the knee is cycled from 0° to 90° of flexion, until arthroscopic visualization confirmed that the marking sutures had fully entered the tibial tunnel. Finally, with the knee flexed to 30°, the shortening loops were alternately tensioned to secure the button against the tibial cortex, thereby completing the final graft fixation. The button loops and the ends of the internal brace sutures on the tibial side were then tied to the button for supplemental reinforcement (Figure 5B).

## DISCUSSION

Younger age, higher body weight, and participation in intense physical activity are significant risk factors for graft failure after ACLR. In contrast, a larger graft diameter is strongly associated with improved rates of return to sports.^10^ Previous studies have established that graft diameters ≥8 mm are correlated with improved survival rates in young patients following ACLR.^11^ However, the suitability of autologous tendon grafts is not always guaranteed. This is particularly relevant for grafts harvested from smaller‐statured female patients, which often yield grafts of insufficient diameter and length. Consequently, obtaining a graft of sufficient diameter often requires additional surgical efforts, which may include harvesting tendons from multiple sources. The all‐inside ACLR technique has gained widespread adoption as a mainstream procedure due to its minimally invasive nature and its capacity to provide robust graft constructs.^12^

This article describes an overlapping suture technique for 4‐strand hamstring graft preparation, which offers distinct advantages. Primarily, the overlapping suture configuration promotes uniform tension distribution across all strands when the graft is tensioned, which may reduce the risk of postoperative graft elongation or failure due to differential viscoelastic creep. Secondly, this method provides high adaptability, allowing surgeons to precisely modulate the final graft diameter (typically 8‐11 mm) and length (usually 58‐65 mm) by adjusting the overlap, thereby optimizing graft dimensions for individual patient anatomy. Furthermore, the standardized whipstitching at the graft ends (20 mm femoral, 18 mm tibial) serves as a clear arthroscopic reference for accurate graft insertion depth, enhancing procedural consistency.

However, several limitations should be acknowledged. The technique possesses a learning curve, as the overlapping suture pattern is more complex than traditional parallel suturing, requiring practice to ensure reproducibility and graft reliability. Although 4‐0 absorbable sutures are used to minimize interference, the potential impact of suture density and tightening force on tendon‐bone healing within the tunnels remains a theoretical concern that necessitates further investigation. This concern also explains our rationale for avoiding larger nonabsorbable sutures. Furthermore, as a technical description, the clinical superiority of this method—particularly regarding long‐term functional outcomes, graft survival rates, and comparison with established techniques—requires validation through prospective comparative studies. Future research should focus on its biomechanical performance, effect on rehabilitation timelines, and patient‐reported outcomes (Table 2).

**TABLE 2:** Advantages and Disadvantages of the Overlap Link Graft

**Advantages**	**Disadvantages**
Maximizing the utilization of autologous tendon resources ensures adequate graft diameter and length suitable for all‐inside ACLR	The tendon weaving technique is technically demanding and more time‐consuming
Overlap link technique ensures uniform tension distribution among all strands under pretension, reducing the potential for postoperative graft loosening while allowing for appropriate adjustment of graft length	Excessive suture placement within the graft may have potential impact on tendon‐bone healing within the tunnels and lead to an increased diameter, complicating graft fixation
Suture bundling at the midportion of the graft reduces the likelihood of midsubstance gaps appearing on postoperative imaging	

ACLR, anterior cruciate ligament reconstruction.

In summary, the 4‐strand hamstring overlap link technique preserves native tendon integrity and ensures a sufficient graft diameter through quadruple folding. It achieves uniform tension distribution across all strands and allows for precise adjustment of the total graft length via the overlapping suture configuration, making it suitable for most all‐inside ACLR scenarios. Nevertheless, further biomechanical studies and long‐term clinical follow‐up are required to evaluate tendon‐bone healing quality and patients’ return‐to‐sport outcomes.

## DISCLOSURES

The authors (Y.Y., C.C., Y.T., D.W., Y‐M.W., Q‐C.Z.) declare that they have no known competing financial interests or personal relationships that could have appeared to influence the work reported in this article.

## FUNDING

This work was supported by grants from the Fundamental Research Funds for the Central Universities (No. WK9110000143) and the Doctor Research Start‐up Fund (No. RC2024046).
